# Early Detection of Stripe Rust in Winter Wheat Using Deep Residual Neural Networks

**DOI:** 10.3389/fpls.2021.469689

**Published:** 2021-03-30

**Authors:** Michael Schirrmann, Niels Landwehr, Antje Giebel, Andreas Garz, Karl-Heinz Dammer

**Affiliations:** ^1^Department of Engineering for Crop Production, Leibniz Institute for Agricultural Engineering and Bioeconomy, Potsdam, Germany; ^2^Research Group “Data Science in Agriculture”, Leibniz Institute for Agricultural Engineering and Bioeconomy, Potsdam, Germany; ^3^Machine Learning Group, Department of Computer Science, University of Potsdam, Potsdam, Germany

**Keywords:** yellow rust, monitoring, deep learning, wheat crops, image recognition, camera sensor, ResNet, smart farming

## Abstract

Stripe rust (Pst) is a major disease of wheat crops leading untreated to severe yield losses. The use of fungicides is often essential to control Pst when sudden outbreaks are imminent. Sensors capable of detecting Pst in wheat crops could optimize the use of fungicides and improve disease monitoring in high-throughput field phenotyping. Now, deep learning provides new tools for image recognition and may pave the way for new camera based sensors that can identify symptoms in early stages of a disease outbreak within the field. The aim of this study was to teach an image classifier to detect Pst symptoms in winter wheat canopies based on a deep residual neural network (ResNet). For this purpose, a large annotation database was created from images taken by a standard RGB camera that was mounted on a platform at a height of 2 m. Images were acquired while the platform was moved over a randomized field experiment with Pst-inoculated and Pst-free plots of winter wheat. The image classifier was trained with 224 × 224 px patches tiled from the original, unprocessed camera images. The image classifier was tested on different stages of the disease outbreak. At patch level the image classifier reached a total accuracy of 90%. To test the image classifier on image level, the image classifier was evaluated with a sliding window using a large striding length of 224 px allowing for fast test performance. At image level, the image classifier reached a total accuracy of 77%. Even in a stage with very low disease spreading (0.5%) at the very beginning of the Pst outbreak, a detection accuracy of 57% was obtained. Still in the initial phase of the Pst outbreak with 2 to 4% of Pst disease spreading, detection accuracy with 76% could be attained. With further optimizations, the image classifier could be implemented in embedded systems and deployed on drones, vehicles or scanning systems for fast mapping of Pst outbreaks.

## Introduction

Stripe rust caused by the fungus *Puccinia striiformis* Westend. f. sp. *tritici* Eriks. (Pst) is one of the major diseases that lead to severe yield losses in wheat crops. Pst possesses a high genetic variability for developing new and often aggressive strains, which led to major epidemic outbreaks in North America and Europe in history (Chen et al., 2014). Under the effect of global warming, this trend is currently worsening because Pst has adapted to warmer conditions promoting its wider global propagation with devastating effects in major wheat-producing areas in China, Northern Africa, the Middle East, and India (Milus et al., 2009; Hovmøller et al., 2010). Today, almost 88% of the global wheat production is susceptible to Pst (Beddow et al., 2015). The use of disease-resisting cultivars is an effective and ecologically feasible way to control Pst (Chen et al., 2014; Park, 2016). However, the farmer may prefer to choose a wheat variety with a less good rating of rust resistance in favor of specific market requirements. In addition, new Pst races are known to appear rapidly that overcome major resistance genes in wheat varieties (Hubbard et al., 2015). Often only the application of fungicides remains the optimal choice for the farmer to control Pst when sudden outbreaks loom immanent. If sensors were available that were able to detect Pst outbreaks reliably in the fields in early development phases, it would help to control and reduce the use of fungicides more efficiently (Tackenberg et al., 2018).

The disease develops in the leaf tissue, but also affects spikes and stems in later stages. Initially, patches of unspecific bleaching become visible on the leaves. Then, long and narrow stripes appear between the leaf veins with a yellow to orange coloration (Chen et al., 2014). They are provoked by densely clinging uredospore pustules. Despite its obvious appearance, early detection is still challenging as only a few leaves will carry these symptoms at the beginning. Yet, each pustule is highly infectious and can produce thousands of spores that may initiate subsequent spreading of the disease over the entire field. Thus, field inspections should be done periodically throughout the season to identify outbreaks in a timely manner. Nevertheless, field inspections are time-consuming and laborious, which limits them to be conducted only punctual in the field and during the season.

A number of research studies investigated the use of hyperspectral sensors to detect patches of the Pst infection on plants. Bravo et al. (2003) and Moshou et al. (2004) showed that hyperspectral measurements in the spectral range between 460 and 900 nm are able to differentiate Pst from healthy wheat plants. Huang et al. (2007) found that the photochemical reflectance index (PRI) has high potential for the identification of Pst from airborne hyperspectral images. A limited upscaling to satellite remote sensing was presented by Yuan et al. (2013). Yet, hyperspectral imaging is costly in terms of investment and data handling. Moreover, discolorations in the plant canopy can be the result of nutrients deficiency, herbicide toxicity, or water deficiency (Singh et al., 2018) and may falsely be identified as Pst when only the reflectance on pixel level but not the form of the disease symptoms is included in the image analysis.

The disease symptoms are readily visible by the human eye and Pst develops a characteristic pattern on the leaf’s surface. Instead of focusing on the spectral domain, the analysis of the spatial association of pixels as shown by color images taken from infected leaf spots may also be promising. This would enable the use of simpler camera systems for Pst detection as intelligent sensors. In this regard, attention for image-based plant classification has been given for using segmentation-based (Nilsback and Zisserman, 2008; Khatra, 2013), morphological-based (Munisami et al., 2015), and texture-based (Wang et al., 2012) as well as the use of local image descriptors (Nilsback and Zisserman, 2010). Based on color image segmentation, Khatra (2013) was able to extract yellow rust from wheat plant images using k-means clustering. Wang et al. (2012) could successfully classify yellow rust by training a one-hidden-layer backpropagating neural network with 50 global features from segmented yellow rust images. Local image descriptors are often used within a multi-stage process, where, first, local features are described on a low level and summarized within a visual vocabulary, then feature vectors are related to the visual vocabulary and these relations are finally used for an image classifier based for example on a support vector machine. Bag of visual words is the best known approach among them (Csurka et al., 2004) and, specifically, for plant and weed detection good results have been obtained (Kazmi and Andersen, 2015; Pflanz et al., 2018).

Recently, object-based image classification has been shifted mainly toward the use of convolutional neural networks (CNN), as originally introduced by LeCun and Bengio (1998). CNNs are able to perform feature extraction and classification in one step, i.e., the filters that extract the features for the classification are directly learned within the network and, thus, avoid the dependence on user-defined implementation of feature extraction. It is hoped, that with the use of CNNs, the image classifier can cope much better with the complexity of cluttered field scenes in order to detect the relevant information. CNNs are inspired by the receptive field as found in the human visual cortex (Kim et al., 2016). They include convolutional and subsampling layers (strided convolution layers), which perform the feature learning, feature extraction and dimension reduction. The feature learning part of the network is then followed by dense layers, which will decide the final class label (Rawat and Wang, 2017). CNNs have recently gained respectable image classification results due to the use in large deep learning architectures (DCNN) such as AlexNET (Krizhevsky et al., 2012) or ResNet (He et al., 2016). Within these DCNNs several convolution and pooling layers are stacked block by block, often integrating additional architectural features serving as dropout layers (Srivastava et al., 2014) or shortcut connections (He et al., 2016). Due to the high performance in image classification and recognition, DCNNs are currently becoming a hot topic for plant phenotyping research as reviewed in Singh et al. (2018). Pawara et al. (2017) compared DCNNs using AlexNET and GoogleNET with classical local image descriptors using a bag of visual words framework for plant classification. They could show that DCNNs clearly outperform bag of visual words on three different plant image datasets. Based on DCNN, Mohanty et al. (2016) developed a plant disease detection model using a large image data set of plant leaves with 38 class labels. The architecture of the models used AlexNET and GoogleNET and reached very high accuracy on the dataset itself, yet medium accuracy with random plant images taken from the web. A smartphone app was subsequently developed. It is based on machine learning algorithms and updated with crowdsourcing data, i.e., users can evaluate plant images by sending them in via their smartphones. It is said to recognize up to 400 plant diseases (Rupavatharam et al., 2018). More specifically focused on wheat diseases, Lu et al. (2017) developed an in-field automatic wheat disease diagnosis system based on modified fully connected network architecture also using smartphone images (Long et al., 2015). They built up an image data set called Wheat Disease Database 2017 (Lu et al., 2017) depicting different wheat diseases including stripe rust with no background covering and used it for training the DCNN. The prediction model was later packed into a real-time mobile app to provide support for agricultural disease diagnosis in the field (Lu et al., 2017).

However, images from smartphones differ because they are already focused on the object of interest. In case of smartphone detection, the user has first to act as a sensor in order to determine that there is a specific symptom on the leaves present (Siricharoen et al., 2016). Our aim is, however, to detect Pst from unobserved imagery that can be collected from camera sensors deployed on drones, vehicles or high-throughput scanning systems. In this manner, a large annotation image data base was collected for Pst with the support from a randomized field trial and trained a ResNet convolutional neural network for classifying images as Pst. The image classifier was constructed under the constraints to be integrated in an online sensor system in the future. This study shows the classification performance in relation with different Pst development stages.

## Materials and Methods

### Field Experiment

**FIGURE 1:**
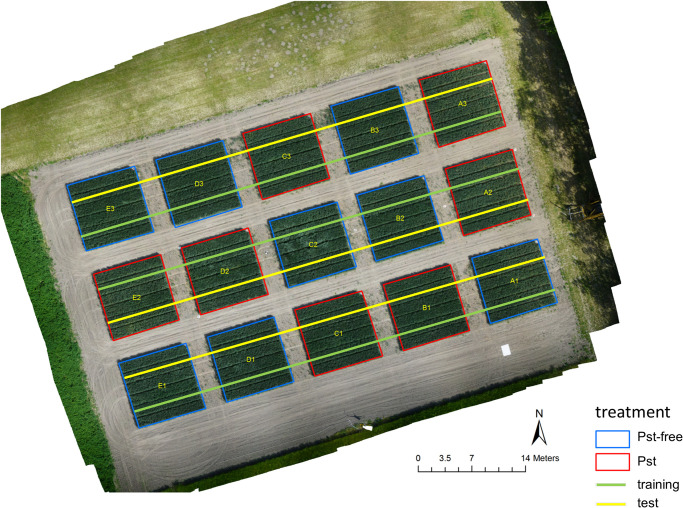
An ortho image generated from a drone in 10 m altitude showing the field experiment at the research station Marquardt (ATB), Potsdam, Germany (52° 28′ 00″ N, 12° 57′ 30″ E). Images were recorded in 2 m altitude along the superimposed lines using a moving equipment carrier.

For the experiments, 6 Pst infested plots (+1) and 6 control plots (+2) of a randomized field trial at the research site Marquardt (ATB), Potsdam, Germany (52° 28′ 00″ N, 12° 57′ 30″ E) were used in the year 2018. The additional plots were originally installed for planning security, but were later used in the experiment. In all plots, winter wheat was sown as the variety Matrix B with a Pst resistance rating of 8 (highly susceptible). The seed row distance was 0.12 m and the seed rate was 350 grains per m^2^. The plots were arranged as shown in Figure 1. Each plot had the dimension of 9 × 9 m and was separated from each other with a distance of 3 m to avoid confusion of management from one plot to the other. Each Pst plot was inoculated starting with April 11, 2018 during cold and calm weather. For inoculation, a spore solution with 2.5 g Pst spores and mixed with 500 ml purified mineral oil was prepared. The spore solution was evenly spread with an Ulva+ hand-held spinning disc sprayer (Micron Group, Bromyard, United Kingdom) over the whole plot. Control plots were treated with the fungicide Osiris^®^ (BASF, Germany) on May 3, 2018 to guarantee that they were free of disease.

Beginning with 20 days after Pst inoculation (dai) on May 3, 2018, the experimental plots were weekly assessed for Pst symptoms occurring on the plants. The Pst assessment scored the percentage of infestation symptoms for the first three top leaf layers of the plants. Pst assessment was conducted at six locations within each plot near the locations where the images were acquired. At each location, 10 wheat plants were selected randomly for the assessment.

### Image Acquisition and Annotation Database

All images were acquired with the DSLM camera ILCE-6000 (Sony, Japan) with an APS-C type sensor chip (23.5 × 15.6 mm) and a 50 mm lens attached (SEL50F18, Sony, Japan). The camera was installed on a boom, which was mounted on an equipment carrier in nadir position. The distance between camera lens and ground was fixed to 2 m. The projected area on the ground was 0.63 × 0.97 m. The equipment carrier was slowly driven through the field experiment while the boom was always holding the camera with 2.25 m distance off the plot boundary in order to prevent edge effects. While moving, the camera was automatically triggered via USB using a wheel sensor installed on the equipment carrier approximately every 1 m. Images were acquired from both sides of each plot (Figure 1). Camera parameters were adjusted to a f-number of 8 or 7.1 and to an exposure time of 1/1000 or 1/800 depending on the ambient conditions. Images were recorded on dai 34, 42, 47, and 56.

After excluding the images that were not recorded within the plots, a total number of 2772 images was recorded along the green line (Figure 1), which were taken to train the image classifier and 2690 images recorded along the yellow line, from which 1800 images were randomly selected both plot and dai specific for testing the image classifier. Thus, a ratio between training and test data of 60 % to 40 % was obtained. All images from the training set and 1/3 of the images of the test set were further split into image patches of the dimension 224×224 px along a regular, non-overlapping grid. These patches were annotated on screen by experts. For this purpose, a stand-alone tool was written in MatLab 2018b (The MathWorks, Inc., United States) to allow a fast and multi-user annotation. The tool presented a patch image on a monitor screen and the expert had to choose whether the plant material shown within the patch was affected by Pst (category Pst) or not (category Pst-free). Each patch was selected randomly from all patches belonging to a randomly selected camera image from a specific dai and plot. The patch annotation was repeated until a maximum of 200 patches for each category, plot, and dai were found from the training set and a maximum of 50 patches for each category, plot, and dai for the test set. In case of less annotations for a specific group, plot, and dai, the procedure was stopped until all images had been examined. In this way, an annotation image database was built for four dates containing in total 17245 annotated patches for training and 3621 for testing. From the training set, 8000 patches were randomly chosen for validation and optimization during the training of the ResNet-18. This set is completely independent from the test set. In the annotation database, for each patch, their appropriate camera images, plots and dais was documented. Next, 20 images per plot and dai were annotated from the remaining 2/3 of the test set (1200 images) for testing the image classifier on the full resolution camera images. The annotation was applied to the entire image and upon recognition of one Pst symptom, the image was labeled as Pst otherwise as Pst-free. The number of all images and patches that were used for training and testing the image classifier were summarized in Table 1. Due to technical reasons such as not continuous speed of the equipment carrier when trespassing the experiment as well as time-specific differences due to unequal distribution of Pst symptoms, an exact balances between the eight combinations of dai, training and test set could not be reached.

**TABLE 1:** Number of images and patches used for training and testing the image classifier displayed according to the acquisition of the imagery in terms of days after inoculation (dai).

Acquisition date	dai	Training images (6000×4000 px)	Training patches (224×224 px)	Test patches (224×224 px)	Test images (6000×4000 px)
15.5.2018	34	647	3273	743	300
23.5.2018	42	816	5058	794	300
28.5.2018	47	717	5012	805	300
6.6.2018	56	592	3902	784	300
All	34–56	2772	17245	3126	1200

Finally, drone imagery was collected from the disease outbreak. For this, aerial images were taken from 10 m altitude for all experimental plots on dai 36, 42, 46, and 58 with the same camera sensor as the images on ground were sampled but with a 16 mm lens attached. Ground resolution was 3.84 mm/px. We used a Quadrocopter (HP-X4-E1200, Hexapilots, Germany) and a flight planning that yielded an image overlap of 80%.

### Deep Residual Network Architecture

Image classification was based on a residual neural network (ResNet), a state-of-the-art deep convolutional neural network architecture for computer vision. The key feature of ResNets is the use of so-called residual blocks in the network architecture (He et al., 2016). A residual block consists of a shortcut connection, which implements the identity function x, and in parallel a stack of convolution layers whose output F(x) is added to the identity mapping to form the output F(x)+x of the residual block. Therefore, the stack of convolution layers only has to learn a residual term that refines the input of the residual block toward the desired output. It has been shown that ResNets are easier to train compared to plain convolutional neural networks that simply stack convolution layers, especially for deep architectures (He et al., 2016). Specifically, the identity mappings enable the direct propagation of information and gradients across multiple layers of the network, leading to better gradient flow and convergence properties (He et al., 2016). The ability to train deeper networks with residual blocks has led to a breakthrough in accuracy for major image recognition benchmarks such as ImageNet (Russakovsky et al., 2015).

**FIGURE 2:**
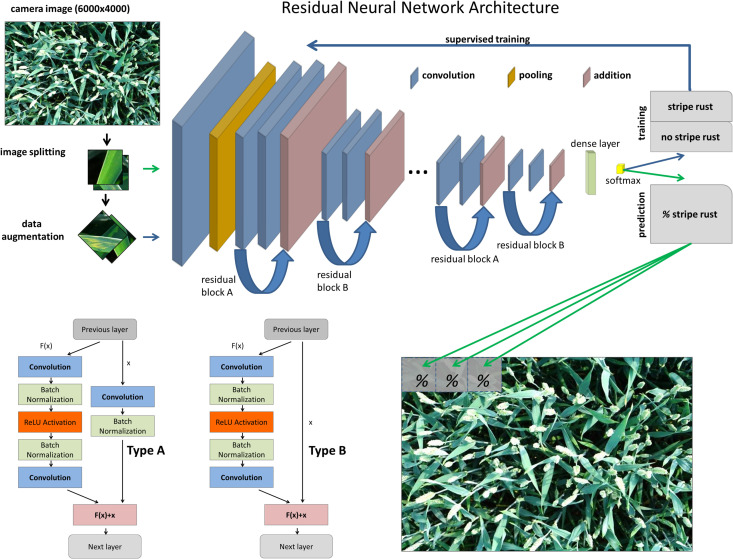
Workflow of the image classification for training and predicting Pst in the full resolution images using the architecture of the ResNet-18 convolutional neural network.

The general ResNet architecture used in this study is depicted in Figure 2. It incorporates two different types of residual blocks (Type A and Type B). The residual mapping F(x) is identical for the two types of blocks and consists of two convolution layers with intermittent batch normalization and ReLU activation functions. Type B follows the original design proposed by He et al. (2016) with an identity mapping for the non-residual branch in the block, while Type A implements a modified version where a single convolution layer is added to the non-residual branch (He et al., 2016II). Type A can be seen as a middle ground between a fully residual and a standard, stacked convolutional block. Specifically, the ResNet-18 architecture was used here, which stacks several residual blocks on top of each other, alternating between Type A and Type B. Directly after the input layer, an initial convolution layer with 64 filters and stride two is followed by a max pooling layer with kernel size two and stride two to reduce the spatial dimension of the input. The main part of the network is comprised of eight residual blocks; alternating between Type A and Type B. The number of filters in the convolution layers of the eight residual blocks is 64 for the first two blocks, 128 for the next two blocks, 256 for the next two blocks, and 512 for the final two blocks. The final convolution layer of every second block reduces the spatial dimension by employing a stride of two, while all other convolution layers in the residual blocks employ stride one. All convolution layers in the network have a kernel size of 3 × 3. The residual blocks are followed by a global average pooling layer and, in the end, one dense layer with softmax activation for binary classification was used. The model is implemented using the Keras library^1^ with Tensorflow backend (Abadi et al., 2015). The full model architecture is shown in Supplementary Figure S1.

### Training the ResNet Model

The ResNet-18 model was trained using the 224 × 224 px training patches from the annotation database along with their associated labels. To increase the number of image patches available for training, the training set was augmented by adding for each original training patch, copies that were rotated (by 90°, 180°, and 270°) and additionally for each rotation angle a copy that was mirrored left-to-right. This procedure yielded eight augmented images patches for each original patch, yielding a total of 137960 image patches fed to the network. The model was trained using the Adam optimizer (Kingma and Ba, 2014) with an initial learning rate of 0.001 and a batch size of 128 for 100 epochs. Training was conducted on a GTX 1080 Ti GPU with 11GB of memory on a server with two Intel Xeon E5-2640 CPUs and 512 GB of main memory running Debian Linux 8.11. After training, the predictive class probabilities returned by the softmax activation in the final layer of the neural network are often not well scaled; specifically, they are often too close to zero or one. Therefore, class probabilities were scaled according to the temperature scaling method described by Guo et al. (2017). While temperature scaling is a monotonic transformation and does not change the classification result, having well-calibrated class probabilities makes it easier to interpret the output of the network (for example, a probability of 0.99 for the Pst class after calibration would indicate high confidence in the prediction). Temperature scaling was performed on the validation patches.

### Applying the ResNet Model

The trained image classifier as described in 2.3 returns a probability for class affiliation of the Pst class, which represents whether the image patch shows signs of Pst infection or not. In the following this probability is referred as the Pst-score of the patch. The goal of this study is to detect stripe rust in the full resolution camera images (6000 × 4000 px). To obtain a prediction for a full image, each image was cut into small patches (224 × 224 px) without overlap across the full image. For each patch, the ResNet-18 model was evaluated and returns a Pst-score. This procedure resulted in 442 image patches for each 6000 × 4000 px full image, and therefore 442 score values for each image.

To classify the full resolution image, a preferably high precision with still acceptable recall is needed. A prediction error of 1% seemed suitable to satisfy both criteria. Thus, the full resolution images was classified as Pst, if only one patch of the 442 patches per image had a Pst-score greater than 0.99.

### Performance Evaluation Metrics

We tested on patch level and image level given the independent test patches and images as described in section “Image Acquisition and Annotation Database.” The following performance criteria were used for evaluation calculated from the resulting true positives (TP), true negatives (TN), false positives (FP), and false negatives (FN):

$$\text{Precision}=\frac{\text{TP}}{\text{TP}+\text{FP}}$$

$$\text{Recall}=\frac{\text{TP}}{\text{TP}+\text{FN}}$$

$$\mathrm{F1}=2x\frac{\mathrm{precision}x\mathrm{recall}}{\text{precision}+\text{recall}}$$

$$\mathrm{False}\mathrm{Positive}\mathrm{Rate}=\frac{\text{FP}}{\text{FP}+\text{TN}}$$

$$\text{Accuracy}=\frac{\text{TP}+\text{TN}}{\text{TP}+\text{TN}+\text{FP}+\text{FN}}$$

On patch level, the standard model threshold of 0.5 was chosen to describe the performance of the model. The relationship between recall and false positive rate (FPR) informs about the power of a binary classification model for a range of thresholds. This curve is called ROC curve (receiver operating characteristic curve). As a measure of the prediction accuracy the AUC (area under the curve) was used to compare the classification models. The value of one is a perfect model, zero means a random prediction.

### Comparison With Drone Imagery

The drone images were photogrammetrically processed with Metashape Professional (Agisoft LLC, Russia, 2019) to produce ortho images of the field experiment. Based on the RGB values, the triangular greenness index (TGI) was calculated, which has a good correlation with photosynthetic activity of dense canopies (Hunt et al., 2013):

$$\text{TGI}={\text{R}}_{\text{green}}-0.39{\text{R}}_{\text{red}}-0.61{\text{R}}_{\text{blue}}$$

TGI maps were used as a qualitative assessment at which point in time the disease outbreak influenced the reflectance signal in such a manner that it was being recognized from typical remote sensing imagery taken from drones.

## Results

### The Course of the Disease Outbreak

**FIGURE 3:**
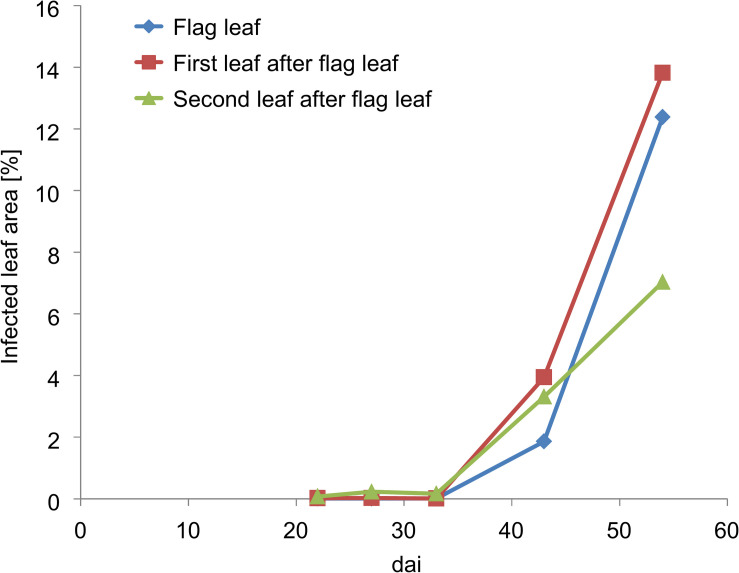
Pst infection development of the upper three leaves according to field scoring values of the inoculated plots. Dai refers to the number of days after inoculation.

First disease symptoms of Pst were determined from dai 28 by field scoring in the lowest leaf layers (Figure 3). These first Pst symptoms occurred very sporadically within the Pst inoculated plots. In most cases, these characteristics were only discolorations due to chlorosis because the growing fungus within the leaf absorbed nutrients and lowered in turn photosynthetic activity. The area of discoloration of the infected leaves was below 0.5%. On dai 42, the Pst infection had developed from the lower leaf layers to the upper leaf layers and the whole canopy sporadically showed Pst characteristics. According to the Pst scoring, around 2% of the upper leaf layer area and around 4% of the lower leaf layer area was infected by Pst at this date. On dai 47, Pst infection rapidly developed upward in the canopy and infected the first leaf layers with an area of around 8 to 10% whereas the development of Pst in the lower leaf layers slowed down and was around 6%. On dai 56, the whole canopy was strongly infected. However, because of the hot and dry spring, the general severity of the disease was rather low.

**FIGURE 4:**
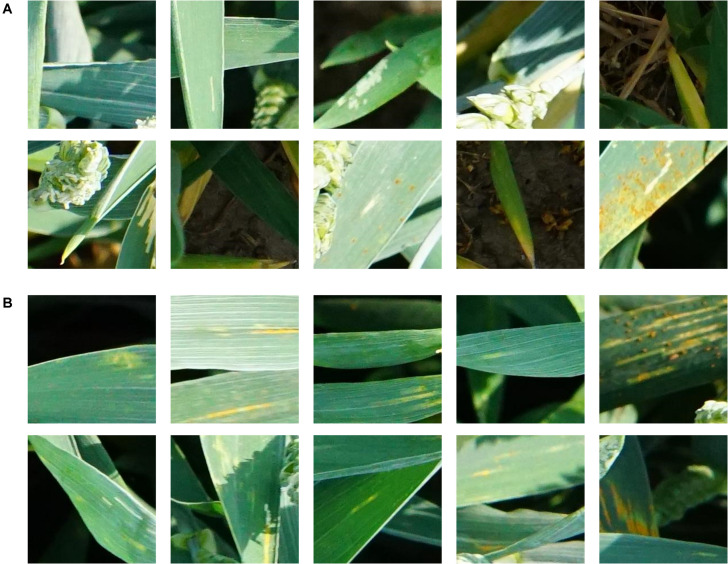
Examples of 224 × 224 px patches showing wheat without **(A)** and with **(B)** Pst symptoms.

Next to Pst characteristics, other weak damages of the leaves occurred in the Pst infected plots. The appearances of some of these symptoms were quite similar to Pst symptoms especially in the earlier stages of disease development. This included slug or snail damage, discolorations of chlorosis not originated by Pst fungi, and symptoms of other fungal diseases such as septoria leaf blotch (*Zymoseptoria tritici*) and leaf rust (*Puccinia triticina)*. In addition to these similarities, the imagery was cluttered with no background separation, leaves could be partly hidden or shadowed, or heavily mixed by many different features such as spikes, leaves, stems and soil. In Figure 4 some of these challenges that the image classifier was faced are summarized.

### Classification Accuracy of Pst at Patch Level

**FIGURE 5:**
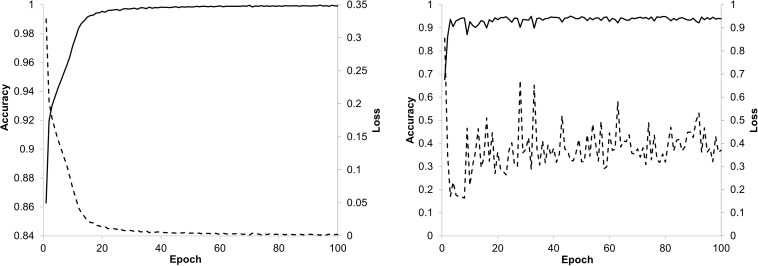
Accuracy and loss trend curves for training **(left)** and validation **(right)** of the ResNet-18 model.

The training of the ResNet-18 model with the 224×224 px image patches from the training set reached a fast convergence after about 20 epochs with the training data and stabilizes after about 40 epochs with the validation data as can be seen from the trend discovered by the accuracy and loss curves in Figure 5. The model showed very good performance during validation with an accuracy between 0.9 and 1.0. The loss in the validation was slightly increased, which may indicate that probabilities were not sufficiently calibrated. That is why a recalibration with temperature scaling was performed on the class probabilities.

**FIGURE 6:**
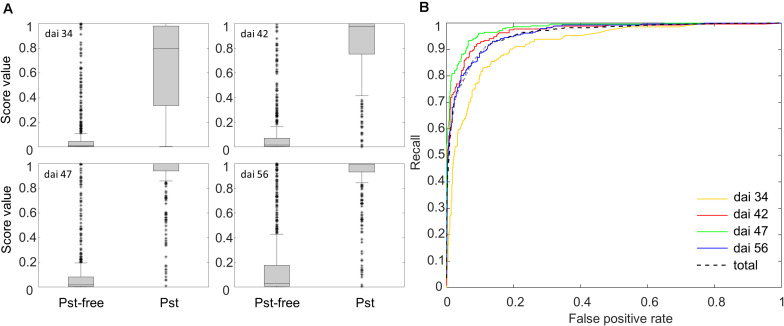
**(A)** Box plots of the Pst-scores evaluated by the Softmax layer of the ResNet-18 model for the group Pst and the group without Pst symptoms calculated from 3126 test patches. **(B)** ROC curves showing the recall and FPR of the ResNet-18 model for classifying 3126 test patches.

After training and scaling of the ResNet-18 model, a prediction of the Pst occurrence was returned as a Pst-score for each new image patch. In Figure 6A, boxplots of those Pst-score values calculated on the basis of the test patches for dai 34, 42, 47, and 56 are shown for the test categories Pst and Pst-free. For all dais, a clear division of the main bulk of score values (non-overlapping boxes) was determined supporting the prospects of a possible image classifier for Pst detection. The tallness of the individual boxes, e.g., the variability of Pst-score values, increased for the Pst-free group whereas decreased for the Pst group with later stages. This might indicate some uncertainty to detect Pst in the initial phase of the outbreak and to detect Pst-free when the disease has fully evolved. Only in the case of dai 34, stronger overlap of the Pst-free and Pst score values occurred in the low Pst-score range as can be seen from the overlapping whiskers. The whiskers signify the lowest or highest value, which are still within the 1.5 times the interquartile range based on the lower or upper quartile. This indicates that the ResNet-18 model over interprets anomalies in the wheat canopy as Pst at the beginning of the outbreak of visual Pst symptoms. However, according to the Wilcoxon test (*p* < 0.001), the Pst-scores of Pst group and Pst-free group were significant different at all dates including dai 34.

**FIGURE 7:**
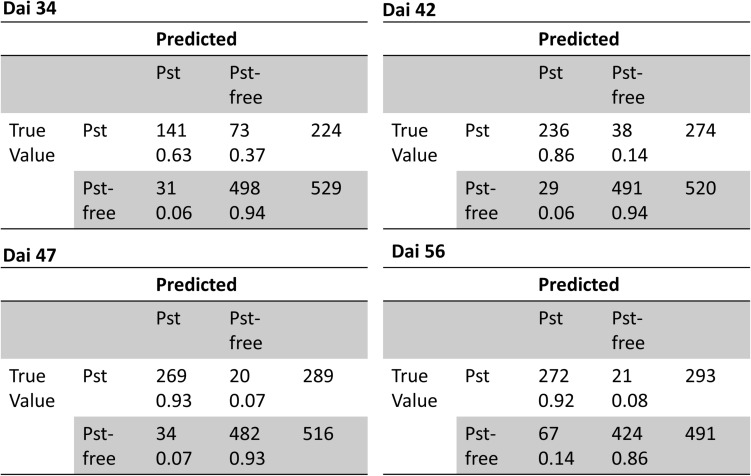
Confusion matrices of classification of the image patches into Pst and non-Pst based on an independent test set (*n* = 3126) for different days after inoculation (dai).

Additionally, ROC curves were calculated from evaluating the test patches. All ROCs exhibited a strong convex curve with their inflection points orientated toward the left-hand upper corner. The AUC values varied between 0.92 and 0.98 showing a good to very good performance for creating a binary classifier for Pst (Figure 6B). The confusion matrices (Figure 7) based on the standard threshold 0.5 and the corresponding performance criteria are given in Table 2 for the individual dais and for all dais pooled. The confusion matrices are depicted for each observed dai, which show directly the number of true positives (TP), false positives (FP), true negatives (TN), and false negatives (FN). The number of TP increased with increasing dai. On dai 34, only 141 from 224 patches (63%) were truly predicted as Pst, on dai 42, it increased to 86% (236 from 274 patches) and on dai 47 it rise above 93% (269 from 289 patches). The characteristics of the TN differs from this. The TN were constant with a very high number for dai 34 to 47 ranging between 93% and 94%, while for dai 56 there was a small decrease to 86%. So, at the beginning of the Pst outbreak on dai 34, the model was more inclined to predict patches as Pst-free, although symptoms of the disease were present. This differs in the latest stage of the disease outbreak (dai 56) when Pst was nearly omnipresent on the leaves. Here, model prediction was slightly reversed. The image classifier had now a higher tendency of predicting patches as Pst although no Pst was present. In general terms, a classification accuracy of greater than 90% was reached with good precision and recall performance, which shows the F1 score of 0.85. For the models of the individual dais, accuracies ranged between 86 and 93%. The best accuracy was reported for the model of dai 47, whereas the weakest model was reported for dai 34. Although the total model performance for precision and recall was balanced, slight variations of the F1 score were observed between the dais.

**TABLE 2:** Classification of patches into Pst and not Pst based on an independent test set (*n* = 3126) for different days after inoculation (dai) and all patches pooled (Total).

dai	TP	TN	FP	FN	ACC	PREC	REC	F1
34	141	498	31	73	0.86	0.82	0.66	0.73
42	236	491	29	38	0.92	0.89	0.86	0.88
47	269	482	34	20	0.93	0.89	0.93	0.91
56	272	424	67	21	0.89	0.80	0.93	0.86
34–56	918	1895	161	152	0.90	0.85	0.86	0.85

*Distribution of counts in true positives (TP), true negatives (TN), false positives (FP) and false negatives (FN) as well as the parameters accuracy (ACC), precision (PREC), recall (REC), and F-measure (F1 score)*

### Classification Accuracy of Pst at Camera Image Level

**FIGURE 8:**
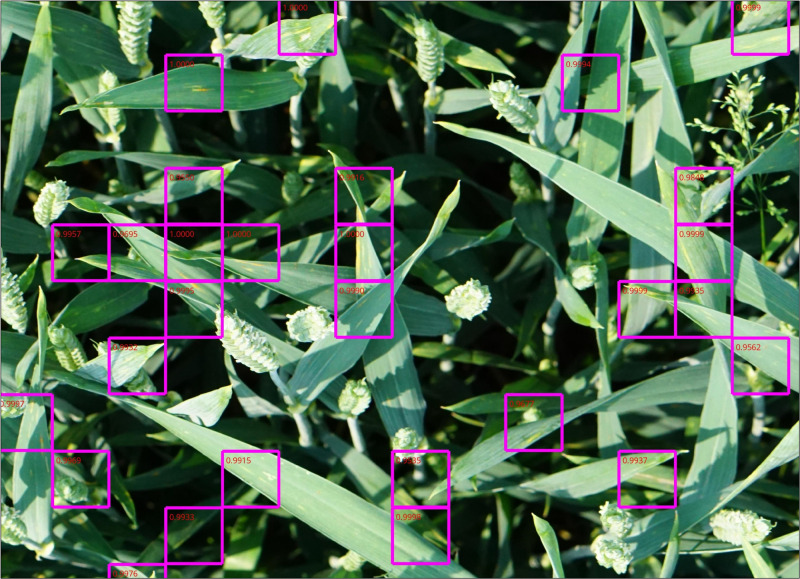
A magnified part of a camera image in a Pst-inoculated plot. The violet rectangles show evaluated patches (224×224 px) given a high Pst-score value (> 0.95) by the ResNet-18 model.

The trained model needed 2.7 s for the full evaluation of one camera image given the designated hardware. In Figure 8, the areas of those patches exceeding the Pst-score of 0.95 were highlighted in a magnified camera image for illustration. It shows that clear Pst characteristics on leaves were easily identified and were associated with a very high Pst-score (> 0.99). In cases where the Pst symptoms were very small, partly covered by objects or unsharpened due to deeper leaf layer position, the Pst-score decreased.

**FIGURE 9:**
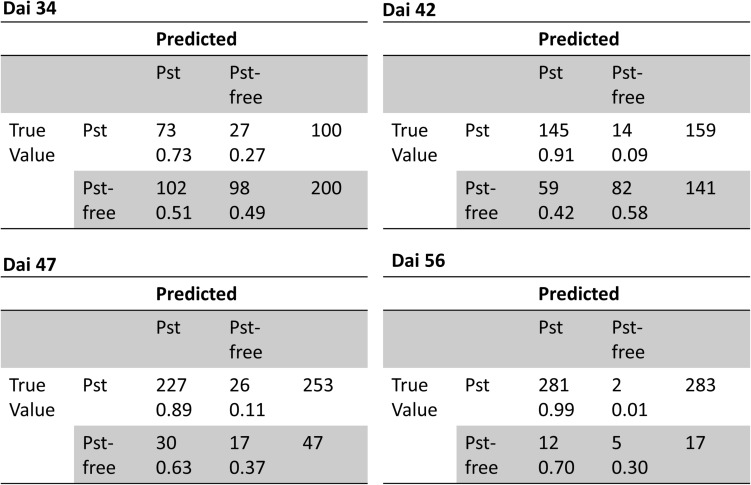
Confusion matrices of classification of full resolution camera images into Pst and not Pst based on an independent test set (*n* = 1200) for different days after inoculation (dai).

The classification was tested on 1200 annotated images and performance results were summarized in Table 3. In total, classification accuracy reached 77% with an F1 score of 0.84 and precision and recall values mostly greater than 0.7. However, there were differences in classification accuracy among the observed dais. The weakest classification accuracy was obtained on dai 34 shortly after the outbreak of the disease. The image classifier reached here an accuracy of 57% with an F1 score of 0.53. Compared to the results at patch level, the F1 score was lower based on a lower precision. According to the confusion matrices shown in Figure 9, the highest FP (27%) occurred for this dai. Because an image contains 442 patches, it seems likely that some false-positive classifications of patches may have occurred mainly in the early stage of disease. One reason could be that the Pst symptoms at this time are in deeper leaf layer position because of early disease stage. On dai 42, classification accuracy rose above 76% with high and well-balanced precision and recall. The confusion matrix for this dai showed a decreased FP (9%) compared to dai 34. This was still in the initial phase of the Pst outbreak, where only 2 to 4% of the leaf area was infected. On the other site, the confusion matrices also reveal some limitations of the full image classifier, since FN was quite high specifically in the later stages of the disease at dai 47 and 56, which means that the classifier detects many images as Pst whereas no Pst is actually present.

**TABLE 3:** Classification of full resolution camera images into Pst and not Pst based on an independent test set (*n* = 1200) for different days after inoculation (dai) and all images pooled (Total).

dai	TP	TN	FP	FN	ACC	PREC	REC	F1
34	73	98	102	27	0.57	0.42	0.73	0.53
42	145	82	59	14	0.76	0.71	0.91	0.80
47	227	17	30	26	0.81	0.88	0.90	0.89
56	281	5	12	2	0.95	0.96	0.99	0.98
34–56	726	202	203	69	0.77	0.78	0.91	0.84

*Distribution of counts in true positives (TP), true negatives (TN), false positives (FP) and false negatives (FN) as well as the parameters accuracy (ACC), precision (PREC), recall (REC), and F-measure (F1 score).*

**FIGURE 10:**
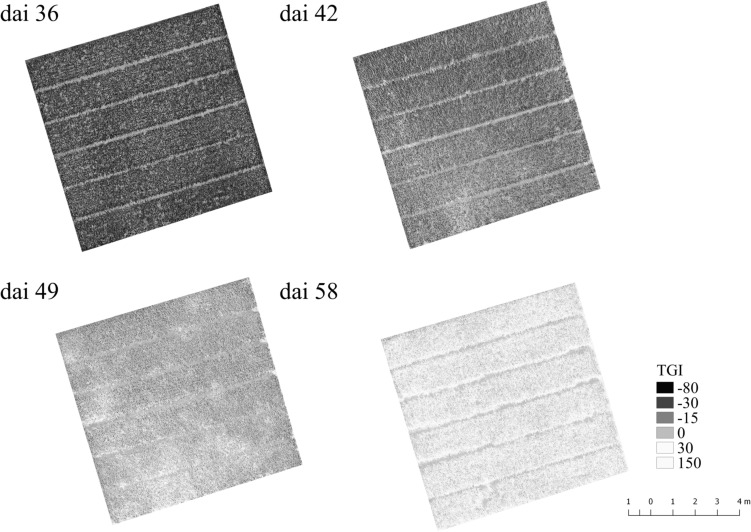
Triangular Greenness Index map (TGI, Hunt et al., 2013) calculated from UAV ortho images taken over the course of the Pst outbreak for a plot with very high Pst occurrence after the outbreak. Dai refers to the number of days after inoculation.

Another interesting point is at which dai, symptoms of Pst can be assumed from typical drone imagery as a remote sensing approach as an alternative method for assessing Pst in the field. In Figure 10, the TGI maps calculated from the drone imagery taken from 10 m altitude are shown for the plot with the strongest disease occurrence over the course of the Pst outbreak. It turns out that effects of the Pst outbreak can only be seen from dai 42 onward and even on dai 42 the Pst nests can only vaguely be assumed because TGI distribution had no structure from which Pst nests were readily recognizable. Without any further information, it would be impossible to assume the Pst infection in this phase. On dai 36, no changes at all were recognizable in the drone imagery. On dai 46, the pattern points to the occurrence of several Pst nests within the plot and on dai 58 the whole plot was infected and TGI values had all changed to higher values. However, this study could show that a detection of Pst with high resolution imagery is possible even under low disease infestation (2 to 4%) with an acceptance rate of 76% by using deep residual neural networks on dai 42. This is even before discolorations in the drone imagery from 10 m altitude could be identified as Pst disease.

## Discussion

The image classifier was trained and tested as if it should be integrated in an online detection system for Pst in the field. Thus, imagery was collected from an equipment carrier passing slowly along the Pst experiment plots with a camera adjusted to nadir position. In no case, we had any control to focus the camera on certain Pst symptoms or to homogenize the background so that an easy segmentation of individual wheat leaves would have been possible. Thus, this study differs from the works of, for example, Mohanty et al. (2016), who successfully classified 38 plant diseases using AlexNet and GoogleNet. However, they used images taken under controlled conditions and performed a segmentation between background and plant leaves before training the image classifier. Also, this study differs from studies that focus on smart phone usage (Lu et al., 2017; Rupavatharam et al., 2018), because in this case the user operates like a monitoring system when directing the smartphone toward the plant anomaly of interest. In our scenario, images were taken fully unobserved, without any pre-selection and Pst infection needed to be identified from them in a high throughput manner.

This posed a lot of challenges to the image classifier. It needed to distinguish the disease symptoms from a highly heterogenic mix of different positioned plant leaves, stalks, spikes and background cluttering. Leaves and its symptoms can be partly covered or shadowed. Image quality may vary due to external dynamics such as wind, which may introduce blurriness in the images. The image classifier was trained from images, which closely adhered to those conditions because it is assumed that this would make the final model more robust for an implementation as an automatic sensor. Technically, the image classifier had to evaluate a new image in a very short time to use it as a sensor. Thus, it was choosen a rather large striding length in the dimension of one patch, e.g., 224 px, which enabled faster evaluation of the camera images. These constraints we put on our image classifier might have reduced classification accuracy to some extent. In contrary, DeChant et al. (2017) produced heat maps of the full resolution images with shorter striding length of 30 px and used an additional CNN that evaluated the heat maps. This improved the classification accuracy for detecting *S. turcica* in the maize canopy; however, they needed several minutes to evaluate one image, which is too long for a future implementation on an online mapping system. In contrary, the patch-combined classification of the whole images seems rather simple. However, most of the information is kept in the patches anyway. One limitation might be that edges of the patches discontinue stripe rust occurrences in the images. This can slightly increase wrong annotation, erroneous learning of the network and faulty evaluation, which leads to a classifier that might be stronger susceptible to errors. On the plus side, the evaluation of the images is fast enough to implement in an online system and no sophisticated post processing of the images is needed. Implementation on an embedded system can solely concentrate on the deep learning network (ResNet-18) itself.

Another source of uncertainty arises from the annotation data base. Even the experts annotating the patches and images on the monitor screen did occasionally run into problems because characteristics of leaves depicted on screen were barely recognizable as Pst or Pst-free. Specifically, lower leaves, shadowed leaves or unsharpened image regions posed problems to the manual annotation on screen. In this study, this error was tried to reduce by using a relatively large annotation size of 17245 patches for training rather than removing the uncertain image data. Obvious errors are only removed by checking of the annotation database. Furthermore, we decided to split the training and test data along the green and yellow line (Figure 1) to maintain equal balance of variability in both data sets. The problem was that in the experiment the Pst infestation severity was different from plot to plot. If the data had been split by plot number, a training and test set with too different Pst occurrences could have been generated, leading to biased results. Transferability to different fields and crops was not tested in this study. Yet, the annotation data base can be extended with more patch-based annotations so that the ResNet-18 model can be retrained to fit other situations as well. Finally, an automatic sensor system that should work unsupervised in the field will always be restricted to a certain angular perspective and viewing range. An expert in the field can hold the leaf in their hands turn it around and look from several angles and distances to estimate the occurrence and degree of Pst infestation. A sensor might easily miss Pst occurrences on the back of the leaves or in lower leaf layers. Yet, an expert is not able to evaluate an entire field but is restricted to only punctual investigations. Even in earlier stages of the disease, single leaves in the higher leaf layers become infected. These can be easily missed by an expert who is not in immediate vicinity in the field but might be recording with an automatic scanning system.

For our approach to work, images are required as input from which the outlines of the symptoms of Pst on infected wheat leaves can be resolved. Thus, near surface images are needed taken only 1 to 3 m from above the wheat canopy. Many phenotyping platforms, vehicle based carriers and low flying drones may meet these requirements. For example, the ETH Zurich has implemented a stationary installation for crop phenotyping for a 1 ha field area with a cable system that enables free movement of sensors over the field experiments (Kirchgessner et al., 2017). This system operates 2 to 5 m above the canopy acquiring high resolution images in an automatic and high-throughput manner. However, this system is stationary and quite expensive to implement. Vehicle-based systems such as conventional tractors or phenomobiles could carry camera based systems collecting high resolution imagery from above the canopy (Schirrmann et al., 2016; Qiu et al., 2019). However, they will be confined to specific tramlines or passing lines in the field so that images will cover only a small section of the field. Drones could collect high resolution imagery as well with much more freedom of movement. However, multiple drones would be needed for complete field coverage at low altitudes and problems such as the downward wind (downwash) of the copters influencing the canopy needed to be solved (Kirchgessner et al., 2017). Higher altitudes of the drone would provide better field coverage, yet, the lower spatial resolution prevents the assessment of individual form characteristic of the Pst disease in the imagery and only integrated reflectance differences can be surmised. This might not be enough. The study of Su et al. (2018) could not recognize Pst in the initial phase of the disease from drone imagery using multispectral camera and testing many different spectral indices. However, they were favorable in discriminating the disease for a classifier when the Pst disease was fully developed using RVI, NDVI and OSAVI. This was corroborated by the TGI maps shown in Figure 8. If the spatial resolution of future drone imagery can be increased, for example, with low flying drone swarms, drones could theoretically use our approach in embedded systems enabling an early warning system for crop diseases.

## Conclusion

Deep residual networks (ResNet-18) proved suitable to identify symptoms of the Pst disease from high resolution imagery of wheat canopies with an overall accuracy of 77% in this study. Detection accuracy was dependent on the disease spreading in the canopy. When the disease was fully developed, detection accuracy was at 95% while during the disease outbreak, with 2 to 4% infected leaf area, detection accuracy was lower at 76%. This was even before the disease developed nests in the plots that could have been recognized from the imagery taken by drones from 10 m altitude. In an even earlier stage of the disease outbreak, with very low Pst spreading of about 0.5% infected leaf area, a detection accuracy of 57% was still obtained. This shows that the stage of the Pst development needs to be taken into account when training and testing suitable image recognition models based on deep learning for disease detection. Furthermore, the model was trained with a focus on an online detection system that can be integrated on a mobile scanner or a drone platform in this study. The only presumption was to use high resolution imagery from above the wheat canopy within the visible spectral range (RGB). Future work should take into account the optimization of the model for integration into embedded systems by still retaining all the properties of the ResNet model. With some adaptations, the prospects are good that the model can be used for real-time mapping of stripe rust allowing for optimizing precise crop protection and field phenotyping.

## Data Availability Statement

The datasets generated for this study are available on request to the corresponding author.

## Author Contributions

MS, K-HD, and AGa: conceptualization. NL, AGi, and MS: conceptualization, ResNet, data analysis, and validation. NL and AGi: programming python and MatLab. AGi and AGa: data collection and annotation. MS: manuscript preparation. K-HD, AGi, AGa, and NL: manuscript editing and contribution. All authors contributed to the article and approved the submitted version.

## Conflict of Interest

The authors declare that the research was conducted in the absence of any commercial or financial relationships that could be construed as a potential conflict of interest.

## Footnotes

1 https://keras.io
