# Supplementary figures and images for: Early Detection of Stripe Rust in Winter Wheat Using Deep Residual Neural Networks
Source: Front Plant Sci. 2021 Mar 30;12:469689. doi: 10.3389/fpls.2021.469689 (PMC8042394; doi:10.3389/fpls.2021.469689)

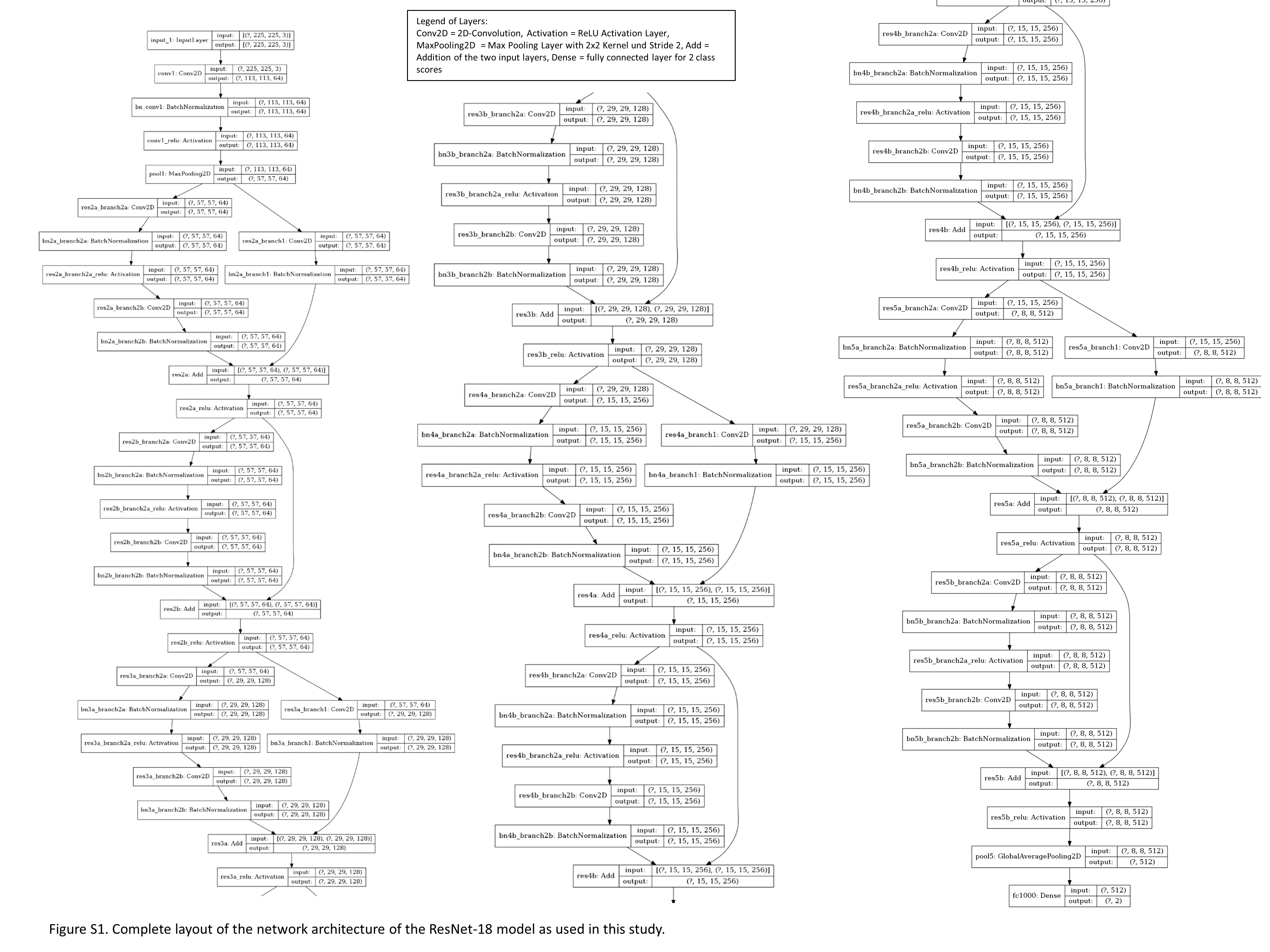

Supplement: Supplementary file 1 [file Image_1.TIF]
